# Mining of Self-Organizing Map Gene-Expression Portraits Reveals Prognostic Stratification of HPV-Positive Head and Neck Squamous Cell Carcinoma

**DOI:** 10.3390/cancers11081057

**Published:** 2019-07-26

**Authors:** Laura D. Locati, Mara S. Serafini, Maria F. Iannò, Andrea Carenzo, Ester Orlandi, Carlo Resteghini, Stefano Cavalieri, Paolo Bossi, Silvana Canevari, Lisa Licitra, Loris De Cecco

**Affiliations:** 1 Head and Neck Medical Oncology Department, Fondazione IRCCS Istituto Nazionale dei Tumori di Milano, 20133 Milan, Italy; 2 Integrated Biology Platform, Department of Applied Research and Technology Development, Fondazione IRCCS Istituto Nazionale dei Tumori di Milano, 20133 Milan, Italy; 3 Radiation Oncology Department, Fondazione IRCCS Istituto Nazionale dei Tumori di Milano, 20133 Milan, Italy; 4 Department of Oncology, University of Milan, 20122 Milan, Italy

**Keywords:** self-organizing map, head and neck cancer, treatment de-escalation, HP, molecular subtypes, tumor microenvironment

## Abstract

Patients (pts) with head and neck squamous cell carcinoma (HNSCC) have different epidemiologic, clinical, and outcome behaviors in relation to human papillomavirus (HPV) infection status, with HPV-positive patients having a 70% reduction in their risk of death. Little is known about the molecular heterogeneity in HPV-related cases. In the present study, we aim to disclose the molecular subtypes with potential biological and clinical relevance. Through a literature review, 11 studies were retrieved with a total of 346 gene-expression data points from HPV-positive HNSCC pts. Meta-analysis and self-organizing map (SOM) approaches were used to disclose relevant meta-gene portraits. Unsupervised consensus clustering provided evidence of three biological subtypes in HPV-positive HNSCC: Cl1, immune-related; Cl2, epithelial–mesenchymal transition-related; Cl3, proliferation-related. This stratification has a prognostic relevance, with Cl1 having the best outcome, Cl2 the worst, and Cl3 an intermediate survival rate. Compared to recent literature, which identified immune and keratinocyte subtypes in HPV-related HNSCC, we confirmed the former and we separated the latter into two clusters with different biological and prognostic characteristics. At present, this paper reports the largest meta-analysis of HPV-positive HNSCC studies and offers a promising molecular subtype classification. Upon further validation, this stratification could improve patient selection and pave the way for the development of a precision medicine therapeutic approach.

## 1. Introduction

Worldwide, head and neck squamous cell carcinoma (HNSCC) affects more than 550,000 patient cases/year with around 380,000 deaths annually [1]. Traditionally, alcohol exposure and tobacco smoking are identified as exogenous risk factors. However, human papillomavirus (HPV) infection, caused predominantly by HPV type 16, is currently recognized as an independent causal factor for the development of HNSCC. Since the 1990s, there was a significant increase in HPV-related HNSCC in western countries, whilst the incidence of HPV-negative HNSCC is globally declining [2,3], in parallel with the decline in tobacco smoking rates. This high incidence of HPV-positive cases establishes HNSCC as one of the most common HPV-related cancers, second only to cervical cancer [4]. Moreover, it is estimated that the annual incidence could increase and eventually surpass the annual incidence of cervical cancer by 2020. Previous epidemiological studies showed that around 25% of all HNSCCs are related to HPV infection, with a tendency for the oropharynx (OPSCC) to be the specific site, compared to infection in other sites (oral cavity, larynx, and hypopharynx) [5]. It is known that HPV-related HNSCC patients have different epidemiologic and clinical behaviors in comparison with HPV-negative HNSCC patients, allowing the identification of HPV-positive HNSCC as a specific distinct disease with peculiar prognostic characteristics [6]. In fact, HPV-positive HNSCC is diagnosed at a younger age than HPV-negative HNSCC, and the five-year survival rate for HPV-positive HNSCC is 60–90% as compared with 20–70% for HPV-negative HNSCC [7], conferring a more favorable prognosis for HPV-positive HNSCC patients. The differences in outcomes between HPV-positive and HPV-negative tumors were already provided, and a multitude of molecular differences comparing HPV-negative and HPV-positive HNSCC patients were reported [8,9,10]. However, a clear biological picture behind their broad diversity is not yet elucidated. Moreover, considering the better prognosis of HPV-positive HNSCC patients compared with their HPV-negative HNSCC counterparts and the median younger age of patients at diagnosis, the question about how to treat HPV-positive patients requires an answer. De-escalation of treatment protocols, for this subgroup of patients, is currently ongoing [11], with the final aim being to reduce the intensity of treatments (both chemoradiation and surgery) and the burden of treatment-related toxicities over the next few years. A further investigation on HPV-related HNSCC is needed. As already reported in the literature, in addition to the diversity of HPV-positive HNSCC compared with HPV-negative HNSCC, it is possible to also observe an intrinsic biological heterogeneity in the HPV-positive HNSCC. In particular, we refer to Keck et al. [12], who identified two different clusters on the basis of their gene expression, and to Zhang et al. [13], who classified these two groups as HPV-positive immune-related (HPV-IMU) and HPV-positive with keratinocyte differentiation (HPV-KRT) HNSCC. Both of these studies had the ability to explore the biology related to HPV infection, unfortunately without showing a significant survival difference.

High-throughput technologies allow the assessment of thousands of features, posing challenges to data analysis. To deal with increased data complexity, researchers apply machine learning approaches to improve biological knowledge via intuitive visualization, even at single-sample resolution. This allows questions, such as biomarker discovery and functional biological information mining, to be addressed. A particular method, self-organizing maps (SOM), provides important benefits including dimension reduction, multidimensional scaling, visualization capabilities over alternative methods such as non-negative matrix factorization, and hierarchical clustering [14]. SOM gained immediate attention in the bioinformatics field, and early microarray studies reported its application [15,16]. Since then, a number of studies on different cancer types proved its robustness [17,18].

In the present analysis, we focused our attention on HPV-positive HNSCC with annotated gene expression data and clinical annotations by exploiting a meta-analysis approach. We applied the SOM machine learning method on a total of 346 HPV-positive tumor samples. This allowed us to dissect the molecular heterogeneity of the disease and to make suggestions for de-escalation treatment.

## 2. Results

### 2.1. Case Material

In order to dissect the molecular heterogeneity in HPV-positive HNSCC, 11 eligible published studies reporting gene expression data were selected for a systematic survey (Table S1, Supplementary Materials). Of these studies, all but one utilized microarray technology for gene expression analysis, and, in the majority of cases, HPV status was assessed with qPCR or HPV genotyping. The resulting meta-analysis dataset, containing 346 samples and 8254 EntrezID genes, was used for the genomic analysis. HPV infection was assessed by p16 immunohistochemistry (IHC) (13 cases, 4%) or DNA or RNA from HPV testing (333, 96%) (Table S1, Supplementary Materials). All the methods used are recognized and utilized in clinical practice [19].

According to the clinical information (Table 1), a male preponderance (83%) and median age of 58.7 years (range, 35–87) were observed, in line with the epidemiological data reported in the literature. The main subsite of origin was the oropharynx (68%), followed by the oral cavity (17%), larynx (6%), and hypopharynx (3%). Stages, assessed following malignant tumor classification system (TNM edition 7, American Joint Committee on Cancer, AJCC), were divided into stages I–II (35), stages III–IV (229), and information not available (82). Locally advanced stages (III–IV) were the most represented (66%), followed by not available (24%) and early stages (I–II; 10%). Survival data were available for 197 cases (57%) and not present for 149 cases (43%). Smoking habits were reported for 245 patients (169 smokers, 76 never smokers), and were unknown for 101 patients (Table 1).

### 2.2. HPV-Positive HNSCC Tumor Clusters: First-Level Self-Organizing Map (SOM) and Unsupervised Clustering Analysis

**Figure 1:**
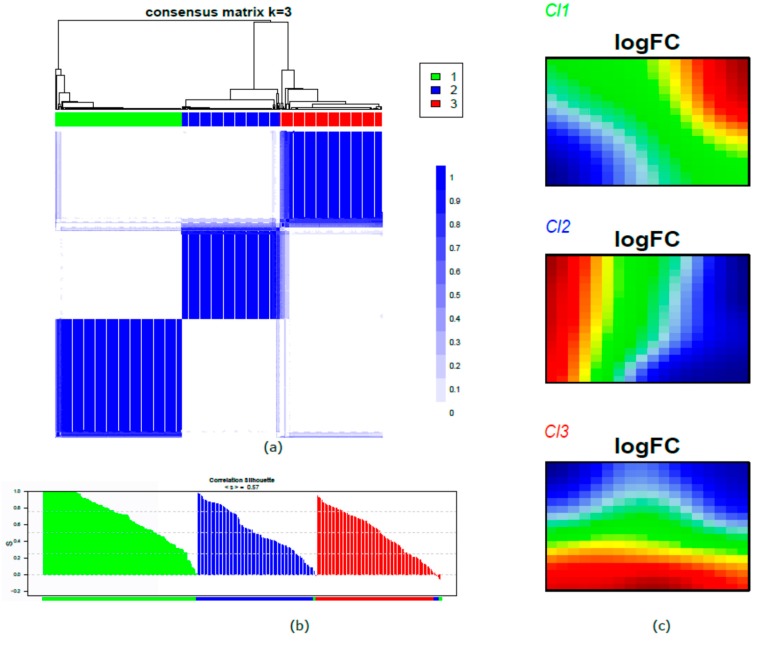
Human papillomavirus (HPV)-positive head and neck squamous cell carcinoma (HNSCC) tumor clusters: first-level self-organizing map (SOM) and unsupervised clustering analysis. (**a**) Consensus matrix heatmap imposing three clusters: Cl1 (*n* = 134; 39%), Cl2 (*n* = 104; 30%), and Cl3 (*n* = 108; 31%). The consensus values are reported in a range from 0 (white, samples that never cluster together) to 1 (blue, samples showing the highest clustering affinity). (**b**) Silhouette plot analysis. The samples are ranked based on silhouette values (S) in each cluster. The heights indicate a strong similarity of the samples within their clusters compared with the samples belonging to other clusters. The colors in the lower bar show the predicted membership by silhouette analysis; the colors correspond to the consensus clustering assignment for all samples with the exception of the seven samples with a negative number but close to 0. (**c**) First level of the SOM gallery of the three clusters with cluster-specific tiles highlighted. The expression patterns are translated into a color code indicating over- and under-expression in a range from red to blue spots, respectively.

We applied the SOM machine learning algorithm to convert the meta-analysis dataset into a matrix of meta-gene expression data. Starting from the 8254 genes, we imposed the log-intensity variation *p*-value < 0.01, and a data matrix of 3498 genes was yielded. These 3498 genes were aggregated in meta-genes (average 10 genes each), resulting in a matrix of 18 × 18 meta-genes. Consensus unsupervised clustering was applied on the meta-gene data, revealing three clusters of samples. The cluster had well-defined boundaries, as shown by the consensus heatmap (Figure 1a). To exclude the existence of under-represented clusters, the consistency of sample assignment was evaluated by silhouette plot analysis. The resulting clustering configuration was appropriate (Figure 1b), since most samples in each cluster had a positive value (average < s >: Cluster 1, Cl1 = 0.68; Cluster 2, Cl2 = 0.53; Cluster 3, Cl3 = 0.48). Only seven samples (two belonging to Cl2 and five belonging to Cl3), corresponding to 2% of the entire case material, had negative values but were in the range between −0.01 and −0.04. These seven samples were assigned by silhouette analysis, as follows: two Cl2 samples to Cl1, three Cl3 samples to Cl2, and three Cl3 samples to Cl1. We assessed the sample size adequacy by estimating the power for the detection of the three clusters; the robustness of the classification was ensured since at least 87% of genes had a power level of 0.9 (Figure S1, Supplementary Materials). By training the SOM algorithm, each sample was portrayed by displaying its molecular fingerprint. The generated subtype SOM images revealed a series of adjacent mosaic tiles coherently over- or under-expressed, and the resulting gallery of SOM portraits was used to intuitively visualize the coherent cluster patterns. In this way, we highlighted cluster-specific tiles in the SOM portraits, independent of the patient’s individuality (Figure 1c).

**Figure 2:**
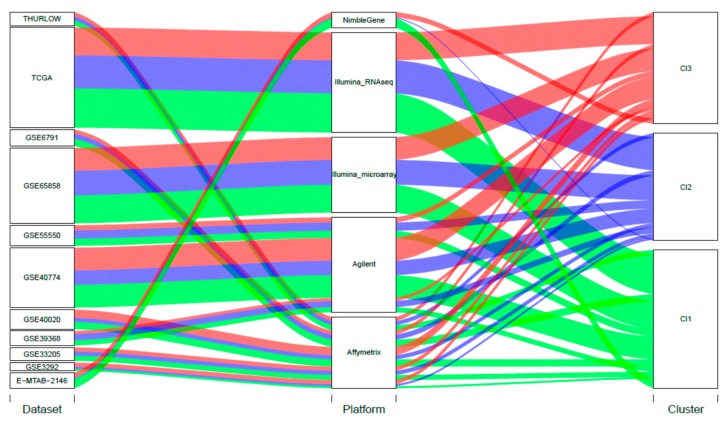
Alluvial diagram. In the diagram, each of the blocks corresponds to the number of features, and the stream fields between the blocks represent changes in the composition of the different blocks. The sizes of the blocks are proportional to the number of samples. We explored the cluster membership taking into account (i) the study of origin of each sample (11 strata); (ii) the different technology platforms used for expression profiling (five strata). Study of the origin: χ^2^ test = 12.08, *p*-value = 0.913; Platform χ^2^ test = 5.93, *p*-value = 0.655.

We also investigated the influence related to technical sources of variability on our findings. An alluvial diagram was used to show the three-cluster membership, based on the study of origin and the platform used for the expression profiling (Figure 2). The percentage of variation, explained by these variables, was investigated compared with the variation associated with the present cluster stratification, and this is summarized in the violin plots (Figure S2, Supplementary Materials). Our findings supported the biological value behind our three-subtype stratification, with a negligible influence of technical covariates.

### 2.3. HPV-Positive HNSCC Cluster Similarity Relationships: Second-Level SOM

The second-level SOM analysis investigated the similarity relationships among the first-level sample SOM portraits.

**Figure 3:**
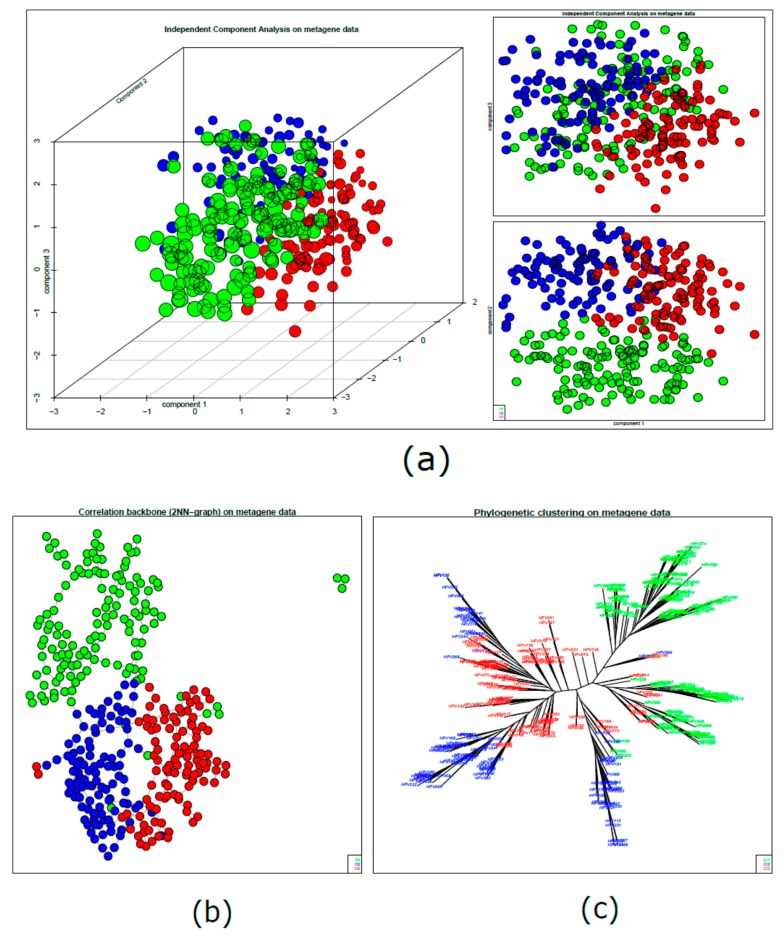
HPV-positive HNSCC cluster similarity relationships: second-level SOM. (**a**) Independent component analysis of meta-gene data. Samples were distributed along the three leading independent components; the plots show the three-dimensional distribution and the projections into the component 1/component 2 (lower panel) and component 1/component 3 (upper panel) dimensions. (**b**) Sample correlation network. The samples are visualized by nodes connected by edges with a backbone structure linking samples with the highest correlation. The similarity between samples is represented by their reciprocal distance; closer nodes have higher similarity and distant nodes have lower similarity. (**c**) Neighbor-joining analysis. The sample similarities are summarized in a phylogenetic tree structure computed using Euclidean distance. The neighbor-joining (NJ) analysis visualizes “bush-like” groups of similar samples by assessing their mutual dissimilarity.

We applied three different sample similarity approaches to estimate the mutual distances among samples, based on metagene expression data and using different metrics and algorithms. The first approach, independent component analysis (ICA), displayed three clusters supporting the identified stratification, although the boundaries between them were not strictly defined (Figure 3a, left panel). Additional information could be retrieved from the three independent components (component 1, component 2, and component 3): the projections onto the component 1/component two axes (Figure 3a, right lower panel) segregated Cluster 1 (Cl1, green spots) from Cl2 and Cl3 (blue and red spots, respectively); however, regions of high density Cl2 and Cl3 showed distinct behavior without clear separation. On the contrary, when the component 1/component three axes were considered, Cl2 and Cl3 were more clearly divided (Figure 3a, right upper panel).

As a second alternative metric, we investigated a correlation network approach: the resulting structure was visualized into a graph to highlight the correlation network (Figure 3b), and it confirmed the presence of a main cluster including Cl1 with few connections to Cl2 and Cl3. 

The third approach exploited a Euclidean distance-based approach through the resolution of neighbor-joining (NJ) clustering, which projects the relationships among samples in phylogenetic trees (Figure 3c). The NJ dendrogram was able to disclose finer details than the previous approaches, and it revealed inherent substructures and their connections in each cluster. By visual inspection, most Cl1 samples were segregated into clearly different branches from Cl2 and Cl3 branches, which, in contrast, appeared tightly correlated.

**Figure 4:**
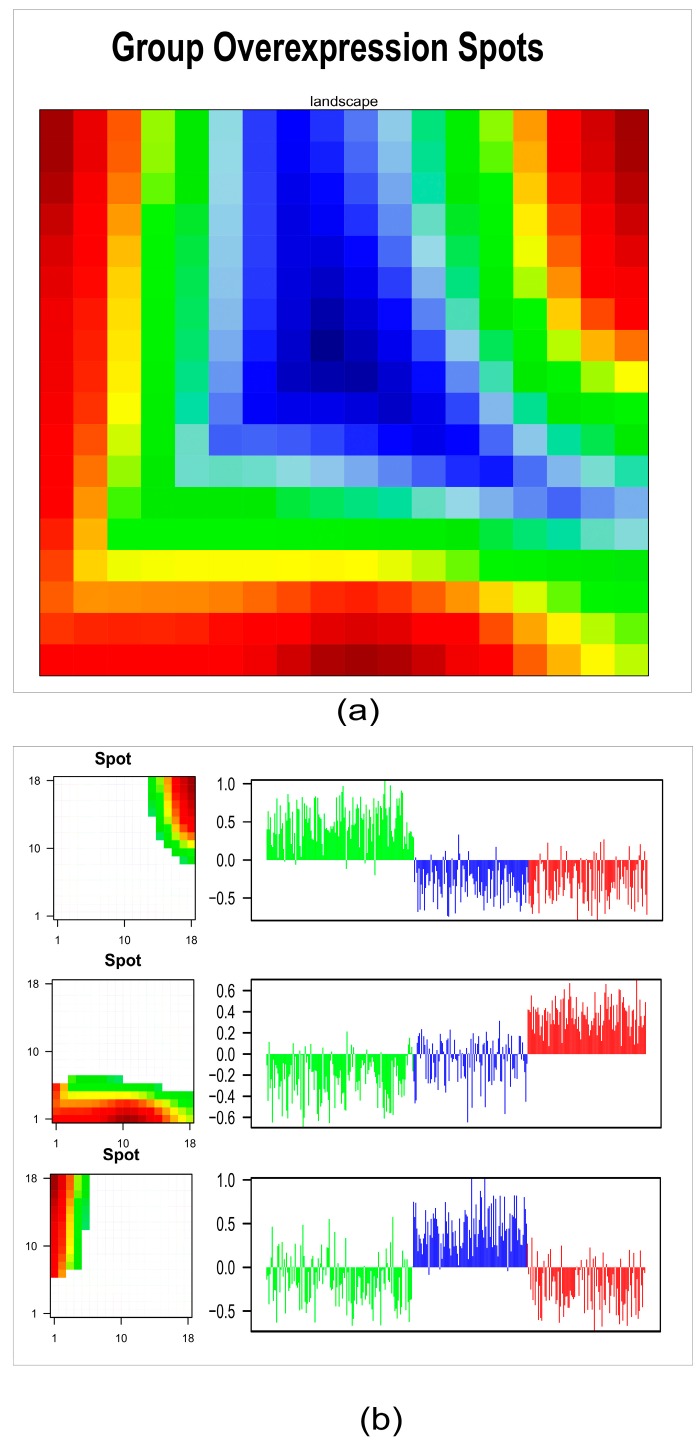
Subtype characterization by group overexpression maps. (**a**) The 18 × 18 map of meta-genes summarizes the expression landscapes over the three subtypes; according to this analysis, co-regulated meta-genes are located in the opposite corners of the map. (**b**) Detailed analysis of metagenes overexpressed in each subtype: map location (left panels) and bar plot of expression intensity (right panels). The bar plot represents the average meta-gene expression of each sample for the selected tiles.

Finally, we investigated the relationship among meta-genes characterizing the three identified subtypes. The process of detection of coherent expression of meta-genes in SOM portraits highlighted specific molecular features for each subtype. Indeed, the resulting map defined three over-expression regions, each of them located in distinct corners of the map. These regions corresponded to SOM clusters of co-regulated meta-genes (Figure 4a). The association of meta-genes to each cluster in precise map locations (left panels) and to a bar plot of expression intensity (right panels) better confirmed and defined the differences between subtypes: 54, 93, and 57 meta-genes had positive correlations with Clusters 1, 2, and 3, respectively (*r* = 0.77, *r* = 0.53, *r* = 0.67) (Figure 4b).

### 2.4. Tumor Microenvironment Landscape

**Figure 5:**
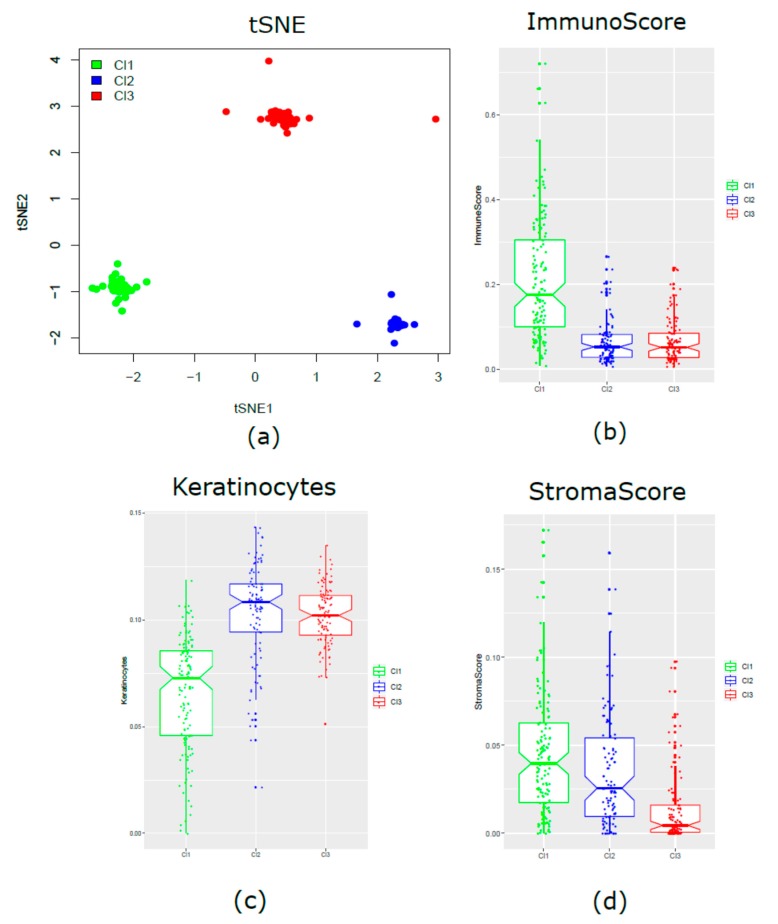
Tumor microenvironment landscape. (**a**) Visualization of the immune and “other cell” infiltrates assessed by xCell. Individual patients are summarized based on two-dimensional coordinates from the t-distributed stochastic neighbor embedding (t-SNE) method. The notched boxplots show the ImmuneScores (*p*-value = 9.9 × 10^−29^) (**b**), keratinocytes scores (*p*-value = 2.03 × 10^−32^) (**c**), and stromal cell infiltrates (*p*-value = 6.3 × 10^−18^) (**d**) split into the three different subtypes.

The xCell tool was applied for the detection and evaluation, if present, of any differences in the three clusters, regarding microenvironment components. According to a dimensionality reduction technique (t-distributed stochastic neighbor embedding, t-SNE), we obtained two-dimensional coordinates that clearly segregated the three molecular clusters. It provided evidence about the existence of unique and defined biological subtypes (Figure 5a). To better disclose the properties of each subtype, the composite scores of immune cells (ImmuneScore), stromal cells (StromaScore), and the score of keratinocytes were calculated. Cl1, compared to Cl2 and Cl3, was characterized by enrichment of immune components *(p*-value = 9.9 × 10^−29^) (Figure 5b) and under-expression of keratinocytes (*p*-value = 2.03 × 10^−32^) (Figure 5c). On the contrary, Cl2 and Cl3 showed similar enrichment in keratinocytes, but a lower immunoscore. Cl2 and Cl3 were clearly separated when compared in terms of stromal components, with Cl3 significantly decreased (*p*-value = 6.3 × 10^−18^) compared with the two other two subtypes (Figure 5d).

### 2.5. Functional Analyses of Subtypes

**Figure 6:**
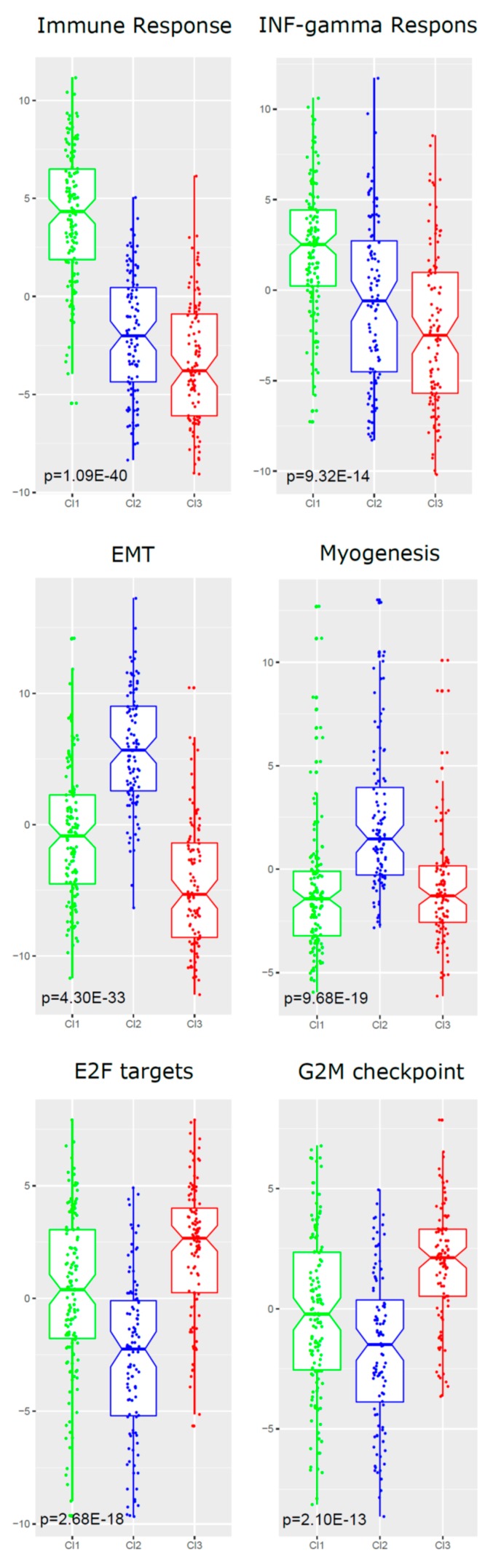
Visualization of the Gene Set Enrichment Analysis (GSEA) functional analysis for each of the three clusters. The boxplots show how the gene set Z score (GSZ) values (depicted in *y*-axis) are distributed within each of the three clusters (Cl1, green; Cl2, blue; Cl3 red). In each row, comparisons of the GSZ score values for the two most enriched hallmark gene sets are shown: for Cl1, over-expression is shown for the “immune response” hallmark (*p*-value 1.09 × 10^−40^) and “interferon (IFN)-gamma response” hallmark (*p*-value = 9.32 × 10^−14^); for Cl2, enrichment is shown in the “epithelial–mesenchymal transition (EMT)” hallmark (*p*-value = 4.30 × 10^−33^) and “myogenesis” hallmark (*p*-value = 9.68 × 10^−19^); for Cl3, over-expression is shown in the “E2F targets” hallmark (*p*-value = 2.68 × 10^−18^) and “G2M checkpoint” (*p*-value 2.10 × 10^−13^). The *p*-values were obtained by means of Kruskal–Wallis tests.

To disclose the biological properties associated with each of the three resulting clusters, further functional characterization was performed using Gene Set Enrichment Analysis (GSEA). GSEA is a method used to test the overrepresentation of genes in gene sets, which are characterized by independent studies. We investigated the “Hallmark” gene set collection representing specific well-defined biological processes. In particular, our analysis provided evidence of a specific enrichment for each cluster. Cl1 showed enrichment in immune-related hallmarks, such as “allograft rejection”, “IFN, interferon gamma”, and “IL6 JAK STAT3 signaling”; Cl2 overexpressed genes related to the hallmarks “epithelial–mesenchymal transition” (EMT), “myogenesis”, and “hypoxia”; Cl3 displayed enrichment in proliferation-related hallmarks, e.g., “E2F targets” and “G2M checkpoint” (Table 2 and Figure 6).

### 2.6. HPV Presence/Integration and Its Association with Clusters

We investigated the association between HPV viral integration and our three clusters, using the data provided by Koneva et al. [20]. Table S2 (Supplementary Materials) shows the contingency table for the TCGA cases analyzed in Koneva et al., reaching a significant association of χ^2^ =12.32 and a *p*-value = 0.00212; the relative presence of HPV integrated cases in each subtype increased in the order Cl1 < Cl3 < Cl2, with relative frequencies of 0.45, 0.77, and 1, respectively. Moreover, we explored the expression of viral genes (E2, E4, and E5). The expression patterns in Cluster 2 are consistent with viral integration. When integrated, the expression of the E2 gene is reduced, since it is truncated along with downstream genes such as E4 and E5 (Figure S3).

### 2.7. Prognostic Values of the Three-Subtype Classification

**Figure 7:**
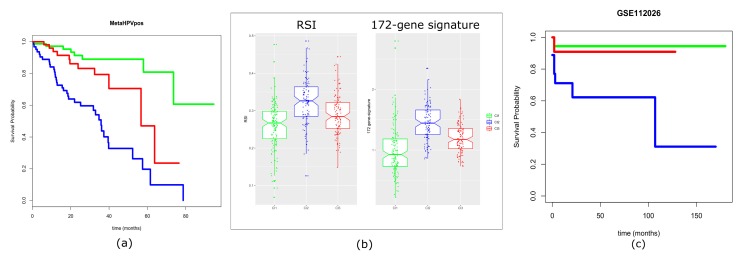
Prognostic evaluation of the three-subtype stratification. (**a**) Survival analysis on the meta-analysis dataset (MetaHPVpos). The 197 cases, entered into the three subtypes (75/134 Cl1 patients; 56/108 Cl2 patients; 66/104 Cl3 patients), were used for the Kaplan–Meier analysis, yielding a log-rank score of *p*-value = 4.76 × 10^−9^. The endpoint was overall survival. (**b**) Gene-signature. Two models were evaluated: (i) radiosensitivity index (RSI), (ii) the 172-gene prognostic model. RSI is directly proportional to radioresistance (high index = radioresistance), while the 172-gene model is directly proportional to the risk of recurrence. Stratification by both signatures reached *p*-value = 8.76 × 10^−13^ and *p*-value = 7.98 × 10^−22^ for the RSI and 172-gene model, respectively. (**c**) Validation on GSE112026. The 47 cases belonging to GSE112026 were stratified based on our three subtypes: 18, 18, and 11 cases were predicted as belonging to Cl1, Cl2, and Cl3, respectively. The cases, entered into the three identified subtypes, were used for the Kaplan–Meier analysis, yielding a *p*-value = 0.0152 (log-rank test).

Due to the robust analysis revealing three distinct HPV-positive HNSCC subtypes, we aimed to investigate their associations with overall survival as the clinical endpoint. Outcome data (i.e., overall survival; OS) were available for 75/134 Cl1 patients, 56/108 Cl2 patients, and 66/104 Cl3 patients, for a total of 197 patients. As depicted in Figure 7a, the results showed a significantly better outcome for Cl1 subtype patients, with a survival probability at 60 months of 0.809, and a worst outcome for Cl3 and Cl2 subtypes, with a survival probability at 60 months of 0.47 and 0.197, respectively (log-rank *p*-value = 4.76 × 10^−9^).

Furthermore, we applied two different gene expression published signatures to the 197 HPV-positive HNSCC patients with available follow-up information: (i) the 172-gene model, a prognostic model for HNSCC [21]; (ii) the radiosensitivity index (RSI) [22], a gene signature developed as a pan-marker of cellular radiosensitivity. In order to assess whether and to what extent the signatures were associated with HPV-related subtypes, we applied the algorithms developed [21,22] to our cohort. The resulting scores were compared to the three-subtype stratification. A significant relationship was found between our stratification and these molecular signatures (Figure 7b). In detail, the Cl1 subtype showed the lowest 172-gene signature related score, meaning that Cl1 has the minimum predicted risk, as confirmed by OS. Furthermore, Cl1 displayed the lowest RSI value, which predicted its radiosensitivity. On the contrary, Cl2 subtype exhibited the highest score in the 172-gene signature, and the maximum RSI score, compared with the other two subtypes, predicting its high risk and intrinsic radioresistance, respectively. The Cl3 subtype showed an “intermediate” behavior, with all three analyses (OS, 172-gene signature score, and RSI).

The clinical relevance of our classification was additionally investigated and associated with the outcome in an external validation dataset. For our analysis, we retrieved the RNA-sequencing (RNA-seq) data of Ando et al. [23], which included 47 HPV-positive oropharyngeal squamous cell carcinomas. With this external validation, we confirmed that the three-subtype stratification provides useful prognostic information. As a matter of fact, better outcomes were associated with patients belonging to Cl1/Cl3 subtypes, and worse outcomes were associated with patients belonging to Cl2 subtype (Figure 7c) (log-rank *p*-value = 0.0152). Finally, we investigated the association between clinal features and our molecular stratification. Table S3 (Supplementary Materials) reports the data related to gender, age, smoking habit, site, and TNM v7 stage. We found a significant association with site having Cl2 a higher percentage of cases other than oropharynx. In addition, due to its potential prognostic role, smoking habit was associated with the three subtypes. There was a trend in the different distribution of the smoking habit with higher percentage of smokers in Cl2. Table S4 (Supplementary Materials) reports the association for Ando’s dataset including gender, age, smoking, Ang et al. (2010) classification system, smoked packs per year, alcohol use, t-stage, and n-stage. We found a significant association with t-stage, having Cl2 cases a higher percentage of T3–4.

## 3. Discussion

Among HNSCCs, the HPV-positive tumors are an independent entity with specific clinical and molecular characteristics. Moreover, inside the HPV-positive subgroup, it is additionally possible to observe an intrinsic heterogeneity, in terms of patients’ outcomes. This assumption questions whether treatment de-intensification could be applied to all HPV-positive HNSCCs. Clinical factors, such as large tumor burden and smoking history, correlate with a worse prognosis, but the biological mechanisms elucidating the complexity of the HPV-positive subgroup are still not fully understood. In the present meta-analysis of transcriptomic data, we applied a rigorous and up-to-date bioinformatics analysis to 346 HPV-positive HNSCCs with published sample data. To the best of our knowledge, this is the largest cohort of HPV-positive HNSCCs analyzed up until now. Specifically, our study identified three tumor subtypes, and it further dissected a population, which was previously divided into only two subgroups by published studies [12,13,24]. In agreement with these findings, we clearly identified an immune-associated cluster (named Cl1 in our analysis). In addition, we stratified the remaining patients (previously described as one “keratinocyte subtype” cluster [13,24]) into two well distinct subtypes with clearly defined biological and prognostic characteristics. The stratification refinement could be attributed not only to the dimension of the analyzed cohort (from two to three times larger than in previous studies), but also to the application of the NJ analysis, which revealed a degree of heterogeneity moving from Cl2 to Cl3 samples with disjointed branches.

In general, HPV-related HNSCCs are known to have better outcomes when compared with HPV-negative HNSCCs [25]. The observed overall survival of our cohort of patients is aligned with the reported prognostic data. However, our analysis displayed a specific prognosis for each cluster, identifying those HPV-positive cases with the best, intermediate, and poorest prognoses. Interestingly, the subtype stratification did not provide evidence of a significant association with smoking habit, but highlighted some specific biological traits for each cluster that could help in interpreting their different outcomes.

Cluster 1 patients exhibited the best outcome at five years and it showed similar behavior to those patients identified as having low-risk HPV-related HNSCC [25]. Additionally, Cl1 was clearly separated from the other two clusters by its high immune score in the xCell analysis, and by upregulation of the hallmarks “IFN, interferon gamma signaling” and “IL6 JAK STAT3 signaling”. The high immune score, associated with a good outcome, could be in agreement with the hypothesis that, in these patients, the immune system plays an important role in the clearance of viral proteins expressed in HPV-positive cancers [26]. Indeed, tumors enriched by the IFN-gamma signature may benefit from immunotherapy [27]. On the contrary, the IL6/JAK/STAT3 pathway hyper-activation is more difficult to interpret in the context of a better prognosis. In fact, IL6/JAK/STAT3 signaling is expected to drive proliferation, survival, and invasiveness of tumor cells, and to suppress the anti-tumor immune response. Overall, we could assume that, in Cl1, the immune infiltrate, as determined by the ImmuneScore, and the high “IFN, interferon gamma signaling” could counterbalance the pro-tumoral action of IL6 JAK STAT3 signaling; however, specific functional assays are necessary to confirm this assumption. Considering the better prognosis and the biological profile, we could hypothesize that Cl1 patients would be the best candidate for de-escalating treatment strategies, even including checkpoint inhibitors.

Cluster 2 exhibited the worst outcomes, and it strongly differed from the other two subtypes by its high stromal score. Essentially, this score reflects fibroblast infiltration, and it frequently leads to deregulation of EMT-inducing factors, EMT upregulation, and hallmark “hypoxia” overexpression. The EMT changes in tumor cells were reported to be linked to the acquisition of aggressive behaviors including (i) increased invasive properties, (ii) resistance to DNA damage, (iii) chemotherapy-induced apoptosis, (iv) immunosuppression, and (v) acquisition of stem-like features [28]. In addition, the increase in the hallmark “hypoxia” is in agreement with the radioresistance detected by RSI [29]. We hypothesize that treatment intensification could be beneficial for these patients. As an example, an accelerated fractionation schedule of radiotherapy should be considered as a strategy to overcome radioresistance.

Cluster 3, characterized by an intermediate outcome compared with the other two clusters, was clearly defined by upregulation of the hallmarks “E2F targets” and “G2M checkpoint”, both associated with increased proliferation. A possible explanation for these data may be the interpretation of boosted proliferation as a result of the integration of the viral genome in the host cell. Moreover, upregulation of the hallmarks “E2F targets” and “G2M checkpoints” is in agreement with the observation that the HPV genome does not encode enzymes necessary for viral replication [26]. Instead, the virus utilizes host cell proteins to replicate its DNA. Therefore, basal cells containing HPV genomes remain active in the pathway related to the cell cycle, also due to Rb degradation. The E2F transcription factor, without Rb function, is free to drive the expression of S-phase genes [26,30]. A first explorative investigation, between the viral integration and our three clusters using data provided by Koneva et al. [20], revealed a significant association between the integration of HPV in the host genome and each of our subtypes in the following order: Cl1 < Cl3 < Cl2 (Figure S2, Supplementary Materials). Despite the analysis being performed on a limited number of samples, Cl2 seemed to be in accordance with cases already described in literature, in which HPV was integrated and viral integration was associated with a poor prognosis [31]. Nevertheless, in this regard, Cl3 shows an intermediate behavior, which may possibly be explained through Nulton discovery [32]. Indeed, HPV infection is described not only as its usual integrated and episomal state but, additionally, as a third state where the viral genome exists as both episomal and integrated states. Anyway, the proposed associations require further evaluation, for not only exploring the HPV state, in terms of integration, episomal, and intermediate states, but also to examine possible target amplification.

Some limitations of this study and some differences with more recent data should be mentioned. Based on the clinical characteristics of the analyzed patients, we observed a relatively high number of missing clinical data (near to 30% in age and stage). The possible explanation for the unavailability of these data could reside in the nature of the studies included in our meta-analysis, which had the biological description of the HPV tumors as a primary endpoint and, accordingly, an inconsistent collection of clinical data was performed.

It is noteworthy that HPV-related tumors in subsites, other than the oropharynx, reached a higher percentage than expected (10%). We hypothesize that this difference could be attributed to the sample collection in the years before the clear prognostic role of HPV infection in oropharynx cancers. In fact, the new TNM staging system (American Joint Committee on Cancer, 8th edition) distinguishes, for the first time, HPV-related from HPV-unrelated oropharynx cancers by stratifying according to p16 expression. The prognostic value for other subsites (i.e., oral cavity, hypopharynx, larynx) other than the oropharynx is still debatable, although a recent review demonstrated a prognostic role for HPV infection in all HNSCC subsites [33].

Considering the prognostic role of our stratification, three subtypes, with different outcomes, were described for the first time. An identified limitation could be the fact that treatment was not systematically recorded, and the overall survival of our case series was poorer than the expected outcome [25]. Moreover, another limitation was identified: the association of subtypes and prognoses should be underscored, although we should highlight that the follow-up was only available for 197 out of 346 (57%) cases. A further bias is related to the differences in treatment techniques used in the last 15 years (e.g., three-dimensional (3D) vs. intensity-modulated radiotherapy; trans-oral robotic surgery, TORS, robotics vs. traditional open surgery).

In conclusion, ongoing trials on de-escalation treatment approaches in HPV-positive HNSCC are based only on HPV status and do not take the contributions of genomics and molecular profiles into consideration [34]. It is conceivable that, upon rigorous validation, our stratification could help develop a “precision treatment approach” based on the genomic profile of HPV-related HNSCC to select patients.

## 4. Materials and Methods

### 4.1. Case Material: Gene Expression and Clinical Data

A survey of gene-expression data on HNSCC (available at 31 August 2018) was accomplished. The cases entered into our study were selected based on the following eligibility criteria: (i) primary lesions of squamous cell carcinoma; (ii) reported HPV status, according to the clinical practice in the reference center; (iii) MIAME (Minimum Information about a Microarray Experiment) [35] complaint data with the availability of raw data deposited on publicly accessible repositories and full gene annotation (Gene Bank accession or EntrezID). After literature revision, there were 11 datasets [12,36,37,38,39,40,41,42,43,44,45]. See Table S1 (Supplementary Materials) for details regarding the datasets including the accession numbers and methods of HPV detection. Raw microarray data were retrieved from the NCBI (National Center for Biotechnology Information) Gene Expression Omnibus (GEO) database [46], ArrayExpress (the EMBL European Bioinformatics Institute, UK) [47], MIAME-Vice [48], and TCGA repositories [49] and were integrated into a unique dataset through a meta-analysis approach, as previously described [50].

In addition, we collected available clinical data related to this case material, comprising age at diagnosis, gender, smoking habits, tumor subsite, stage, and overall survival.

For validation purposes, we retrieved the data from Ando et al. [23], which are publicly available on the GEO repository (identifier (ID): GSE112026). A cohort of 47 primary tumor tissues with HPV-related oropharyngeal squamous cell carcinoma was collected for RNA-seq analysis and microdissected to yield at least 80% tumor purity. HPV tumor status was confirmed by in situ hybridization for high-risk HPV subtypes or p16 immunohistochemistry. According to the TCGA RSEM (RNA-Seq by Expectation Maximization) pipeline, RNA-seq data were processed using RSEM version 1.2.9 and upper quartile normalization. For class prediction purposes, analyses were performed through R-based BRB-ArrayTools software (version 3.5.0) developed by Richard Simon and the BRB-ArrayTools development team [51]. A class prediction method based on a supervised learning method was applied for classifying GSE112026 cases. Prediction was based on the support vector machine (SVM) method by incorporating genes at the univariate significance level (α = 0.001) in a binary tree classification framework, which was chosen due to its ability to classify more than two classes. SVM is specifically designed to address binary classification; however, it can be adapted to handle multi-class classification by building a sequence of binary classifiers. The prediction error of the binary tree classifier was estimated by the leave-one-out cross-validation method.

### 4.2. Data Preprocessing for Meta-Analysis Dataset Generation

The selected studies were analyzed with four platforms, including three microarray platforms (Affymetrix, Agilent, and Nimblegene) and one RNA-seq (Illumina). For Affymetrix data, signal intensities were normalized within each individual dataset using a robust multi-array average (RMA) tool. For Agilent data, the normexp background correction and loess normalization were used for two-channel arrays, while quantile normalization procedures were applied to the probe-level data. For Illumina microarray data, quantile normalization was applied. For RNA-seq data, TCGA level 3 files were downloaded along with the clinical annotations and used for the analysis. The redundancy of probes mapping the same EntrezID was removed by selecting the probe with the highest variance among multiple probe-sets by identifying the same gene; collapse was performed using WGCNA package 1.63 (function: *collapseRows*) and the “*maxRowVariance*” method [52]. To reduce the likelihood of systemic non-biological technical experimental biases among data from different platforms, after log2 transformation, the ComBat algorithm was applied [53]. Then, the expression value of each gene was averaged over all samples of our data matrix, converting the expression data into the change in log-expression (Δ*e_i,m_*) of gene *i* in sample *m*; Δ*e_i,m_* = 0 implies an expression level according to its mean value, while a relative positive or negative value refers to over- or under-expression, respectively, according to the mean gene expression.

### 4.3. Tumor Clusters: First-Level SOM

The Δ*e_i,m_* data matrix was used to train a SOM, an unsupervised machine learning method based on the artificial neural network, enabling the dimensionality reduction of complex data structures of size N × M (N: number of genes; M: number of samples) to K × M (K: number of meta-genes), where K << N, promoting the discovery of qualitative relationships among samples [54]. Each meta-gene represents a cluster of genes sharing similar expression profiles and was selected by an interactive machine learning process by SOM; the process was trained until the meta-genes captured the entire range of expression patterns present in the data matrix. SOM algorithm data analysis and landscape visualization were performed using the “*oposSOM*” R package (version 1.18.0) [55], which uses the “som” R package [56]. A statistical significance criterion based on expression variance was applied to discard the non-informative features in our data matrix through the BRB-ArrayTools developed by Dr. Richard Simon and the BRB-ArrayTools Development Team [57]. The procedure assigns each input gene measured in M samples into a meta-gene of the same length, and each gene is included in a meta-gene, Δ*e_i,m_^meta^*, of closest similarity established by the Euclidian distance. The meta-genes are organized in a two-dimensional grid of K = x × y tiles with the most similar expression profiles of meta-genes adjacent each to another, while the dissimilar ones are more distant. In the present study, we adopted a tile size with an average of n_k_ ≈ 10 genes per meta-gene, corresponding to a two-dimensional grid of size K = 18 × 18 meta-genes with square topology and the Gaussian neighborhood function [14]. The meta-genes were normalized to fit into the range −1 ≤ *e_i,m_^meta^* ≤ 1 and coded by a color scale from blue (low expression) to red (high expression).

### 4.4. Tumor Clusters: Unsupervised Clustering Analysis

The R-package “*ConsensusClusterPlus*” [58] was applied to portion the samples into molecular coherent subtypes. The meta-data Δ*e_i,m_^meta^* were used as input for unsupervised class identification using partition around medoids (PAM) clustering with 1-Pearson correlation as the distance matrix. The PAM algorithm [59] is similar to the K-means algorithm, with both being partitional algorithms that split the dataset into clusters and try to minimize the error. However, while K-means works with centroids, which are artificially created entities that are representative of each cluster, PAM chooses real data-points as cluster centers. An unsupervised clustering procedure was applied to the data through 1000 re-sampling interactions by randomly selecting a fraction of the samples. Cluster numbers ranging from 2 to 10 were tested, and the empirical cumulative distribution function (CDF) and delta area plots displaying consensus distributions were assessed to identify the number of clusters giving maximum stability with a negligible increase in the CDF area [60]. To estimate the accuracy of the classification, the silhouette correlation width values were calculated for all samples (R-package: “*oposSOM*”), providing a graphical representation of how well the samples lay within their assigned cluster. The silhouette values ranged from +1 to −1, indicating the degree of similarity of a sample to the assigned cluster (cohesion) or to other clusters (separation). The evaluation of sample size adequacy of the identified clusters was assessed according to Warnes and Liu (R-package: “*ssize*”) [61] and computed by imposing the type I error rate (false discovery rate, FDR), α = 0.05, and a minimum effect size (log fold-change) of Δ = 1. Cluster-specific portraits represent the mean value of each meta-gene of the samples belonging to the cluster in detail. The portraits are depicted in a log (fold-change) scale where the fold-change is the expression difference compared with the mean expression in all samples. To ascertain to what degree technical variability (i.e., study of origin and platform) affects our subtype clustering analysis, we used the “alluvial diagram”, a variant of the parallel coordinates plot that is helpful for exploring categorical data by grouping them into flows that can easily be traced in the diagram [62]. The plots were generated using the R-package “*alluvial*”. In addition, we used a linear mixed model to quantify the extent of technical variability in each sample through the “*variancePartition*” R package [63]. To visualize the contribution of each variable, violin plots were depicted to show the trend and rank the distribution of variance explained by each variable across all genes. The plots summarize the results in terms of the percentage of variance explained.

### 4.5. Cluster Similarity Relationships: Second-Level SOM Cartography

Second-level SOM analysis aims to address the issue of similarity relationships among groups of samples. It estimates the hierarchy of similarities and mutual distances based on the expression of meta-genes, and it provides improved visualization and representativeness of the results. To infer the main structures present in our data, we applied three approaches for computing the distance metrics.

Independent component analysis (ICA) [64] was applied to the SOM meta-genes using the “*fastICA*” R package [65], a method based on the covariance matrix assessed by Pearson’s correlation to decompose the input meta-genes into independent and non-Gaussian components in order to ensure that each one is statistically as independent from the others as possible.

The correlation backbone through a two-nearest-neighbor graph is a correlation network approach where Pearson correlations are computed between all pairwise combinations of samples, and their structures are visualized in a graph.

The NJ algorithm (“*ape*” R package [66]) is a distance-based method offering phylogenetic tree reconstruction where similarity trees are defined between samples into an Euclidian space, allowing “bush-like clusters” displaying mutual dissimilarity to be revealed [67].

To visualize the main meta-genes related to subtype stratification, we assessed the group over-expression spots. We exploited SOM portraits by detection of the coherent expression of meta-genes. Using group overexpression maps, we linked selected meta-genes (correlation with *r* > 0.5) in different regions of the SOM with groups of samples. The group overexpression portrait was calculated as the mean map profile by averaging the meta-gene expression over the three subtypes. To identify the over-expression tiles, a 98th percentile criterion was applied to the meta-gene expression SOM training aggregate meta-genes with similar profiles in the adjacent neighbored tiles of the map. These tiles’ profiles grouped over-expressed (or under-expressed) samples that differed from the others. The samples belonging to each subtype were summarized in an average representative portrait. The mining of biological functions from SOM portraits was performed using “*oposSOM*” R package (version 1.18.0)**.**

### 4.6. Tumor Microenvironment Landscape

To evaluate the heterogeneity in the tumor microenvironment, the immune, stromal, and other cell components were inferred by an in silico approach using the xCell tool [68,69]. This approach enables the assessment of 64 cell types using the bulk gene expression profiles of the tumors as input and comparing them across samples, as described by the authors of Reference [69]. The tool outputs include the transformed xCell scores for the immune, stromal, and other cell types. The adjusted ImmuneScore included 10 populations (B-cells, CD4+ T-cells, CD8+ T-cells, DC, eosinophils, macrophages, monocytes, mast cells, neutrophils, and NK cells) and StromaScore 3 populations (adipocytes, endothelial cells, fibroblasts). In addition, to identify potential keratinocyte differentiation, the xCell score for keratynocytes was computed. To visualize the cellular heterogeneity of the clusters, we applied a dimension reduction method by t-distributed stochastic neighbor embedding (t-SNE) using the “*Rtsne*” package [70], which projected the cell type enrichment scores onto two-dimensional axes [71]. We presented the scores of each subtype in notched boxplots using the “*ggplot2*” R package. Notch boxplots display a confidence interval around the median based on the median ±1.58 × IQR (interquartile range) /sqrt(n). They are useful graphs for comparing groups of samples, because an absence in notch overlapping provides strong visual evidence that the medians differ. The *p*-values were calculated by the Kruskal–Wallis test, a nonparametric test that compares the means among three or more groups, as in our subtype classification.

### 4.7. Functional Analyses

To disclose the biological functional properties associated with the proposed molecular subtypes, gene set analysis was applied. This approach estimates gene set over-representation (probability of finding genes in a list compared to their random appearance) and over-expression (difference in expression compared to the mean expression over the samples). The gene sets were defined from a priori knowledge from independent studies and they were summarized in a list of genes specifically related to molecular pattern/biological function. A large collection of gene sets was retrieved from the Gene Set Enrichment Analysis (GSEA Broad Institute; software.broadinstitute.org/gsea/) and the Molecular Signatures Database (MSigDB) repository, including 50 hallmark gene sets. We used the gene set Z-score (GSZ) to summarize the profile of a gene set across all samples [72]. GSZ is a Z-score function that merges both over-representation and over-expression features from a gene set to give a defined gene set and provides a representative score of the gene set for each sample. Boxplots were generated using the “*ggplot2*” R package with the notched boxplot function (see Section 4.6).

### 4.8. Analysis of Viral Presence/Integration and Its Association with Clusters

The association between viral integration and our subtype stratification was investigated using the results provided by Koneva et al. [20]. Based on TCGA RNA-seq data and exploiting VirusSeq software [73], they detected known virus strains and identified the integration sites. Thus, the authors disclosed the HPV integration status of 65 TCGA cases present in our meta-analysis and assessed viral gene expression (E2, E4, and E5). We investigated the relative presence of integrated HPV cases defined as integrated cases/(integrated + non-integrated cases) in each subtype, and significance was calculated by the χ^2^ test. Counts per million (CPM) were retrieved from Koneva et al. and transformed into the log scale by log_2_(CPM + 1) [74]. Associations with viral gene expression were visualized by a heatmap. The samples were ranked by the Gene Set Variation Analysis (GSVA [75]) based on the three viral genes. GSVA was used to estimate the variation of a gene set over the samples in an unsupervised manner. The *p*-values were calculated by the Kruskal–Wallis test.

### 4.9. Evaluation of Prognostic Signatures

Statistical analysis was performed using R (version 3.5.1) [76] and Bioconductor (release 3.7) [77]. Survival curves were assessed according to the Kaplan–Meier method, and overall survival was used as the endpoint. Differences between the subtypes were assessed using the log-rank test and R package “*survival*”. Two signatures were evaluated: (i) the 172-gene prognostic model [21]; (ii) the radiosensitivity index (RSI) [22]. The list of genes and the algorithm used for model assessment were retrieved from the original papers.

## Figures and Tables

**Table 1:** Demographic and clinical data of the head and neck squamous cell carcinoma (HNSCC) human papillomavirus (HPV)-positive patients entered in the meta-analysis.

Characteristics	No.	%
**Age, years**		
(median; range)	57 (35–87)	77%
Not available	78	23%
**Gender** (male:female ratio)	287/59	83%/17%
**Subsite**		
Oropharynx	235	68%
Oral cavity	59	17%
Larynx	20	6%
Hypopharynx	10	3%
Not available	22	6%
**Stage according to TNM edition 7**		
Stage I–II	35	10%
Stage III–IV	229	66%
Not available	82	24%
**Smoking**		
Smoker	169	49%
Not smoker	76	22%
Not available	101	29%
**Availability of follow-up data**		
Yes	197	57%
No	149	43%
**Total**	**346**	100%

**Table 2:** Gene-sets significantly up-regulated in each cluster.

Gene-set ID	HALLMARK Gene-Set Name	Genes ^a^	NES ^b^	Nom *p*-Value	FDR q-val
**Cl1 vs. Cl2 and Cl3**					
GS-1	***ALLOGRAFT REJECTION (immune resp)***	130	2.89	<0.00001	<0.00001
GS-2	***INTERFERON GAMMA RESPONSE***	151	2.18	<0.00001	<0.00001
GS-3	***IL6 JAK STAT3 SIGNALING***	60	1.94	<0.00001	<0.00001
GS-4	***INFLAMMATORY RESPONSE***	132	1.76	<0.00001	0.0018
GS-5	***KRAS SIGNALING UP***	114	1.75	<0.00001	0.0019
**Cl2 vs. Cl1 and Cl3**					
GS-1	***EPITHELIAL MESENCHYMAL TRANSITION***	140	3.01	<0.00001	<0.00001
GS-2	***MYOGENESIS***	119	2.42	<0.00001	<0.00001
GS-3	***COAGULATION***	77	2.23	<0.00001	<0.00001
GS-4	***ANGIOGENESIS***	19	2.02	<0.00001	<0.00001
GS-5	***HYPOXIA***	133	1.90	<0.00001	<0.00001
GS-6	***HEDGEHOG SIGNALING***	17	1.89	0.0020	<0.00001
GS-7	***UV RESPONSE DN***	97	1.78	<0.00001	0.0020
GS-8	***APICAL JUNCTION***	137	1.78	<0.00001	0.0020
**Cl3 vs. Cl1 and Cl2**					
GS-1	***E2F TARGETS***	143	2.56	<0.00001	<0.00001
GS-2	***G2M CHECKPOINT***	150	2.24	<0.00001	0.0020

GS: geneset; thresholds: FDR ≤ 0.005; NES≥1.75, ^a^ Number of total genes present in the geneset, ^b^ NES = normalized enrichment score.

## Supplementary Materials

Supplementary materials can be found at https://www.mdpi.com/2072-6694/11/8/1057/s1: Figure S1: Estimation of sample size adequacy. The relationship between the genes in our data matrix and power to detect a sample size defined by our three-subtype stratification (Cl1 = 134; Cl2 = 104; Cl3 = 108) is shown in the plots. The percentage of genes achieving a power level of at least 0.9 is displayed by the red bars and was calculated by performing a pairwise comparison between subtypes; Figure S2: Violin and boxplot of the percent variation in gene expression explained by the study of origin, platform, and our three-cluster stratification. Median percentage variation explained: cluster = 50.4%; study <1%; platform <1%; Figure S3: Expression heatmap of viral genes. The expression values for HPV E2, E4, and E5 in the TCGA cases were retrieved from Koneva et al. For visualization purposes, samples were ranked based on Gene Set Variation Analysis (GSVA) from low to high viral gene enrichment. The membership of each TCGA sample is depicted in the bar below the heatmap. Low E2, E4, and E5 expression was found in Cl2 cases compared with Cl1 and Cl3 (*p*-value = 0.00156, *p*-value = 0.00204, and *p*-value = 0.00147 determined by Kruskal–Wallis Tests, respectively); Table S1: List of used datasets. Dataset name, platform used, provider, technology, repository (included websource), number of samples, assignment to the three clusters, and methods of HPV detection are detailed in the table for each of the 11 sources utilized; Table S2: Contingency table for TCGA HPV cases annotated for HPV integration status by Koneva et al; Table S3: Association to clinical parameters in the meta-analysis dataset. The table includes the evaluation of the following clinical parameters: (i) gender; (ii) age; (iii) smoking habit (current or former smokers vs. never smoke); (iv) site (oropharynx vs. other sites); (v) stage. *p*-values by χ^2^ test, with the exception for age determined by Kruskal–Wallis Tests; Table S4: Association to clinical parameters in the validation dataset (GSE112026). The table includes the evaluation of the following clinical parameters: (i) gender; (ii) age; (iii) smoking, (iv) Ang et al. (2010) classification system; (v) smoked packs per year; (vi) alcohol use; (vii) t-stage; (viii) n-stage. *p*-values by χ^2^ test, with the exception for age determined by Kruskal–Wallis Tests.
