# Supplementary figures and images for: Mining of Self-Organizing Map Gene-Expression Portraits Reveals Prognostic Stratification of HPV-Positive Head and Neck Squamous Cell Carcinoma
Source: Cancers (Basel). 2019 Jul 26;11(8):1057. doi: 10.3390/cancers11081057 (PMC6721309; doi:10.3390/cancers11081057)

CI1/CI2

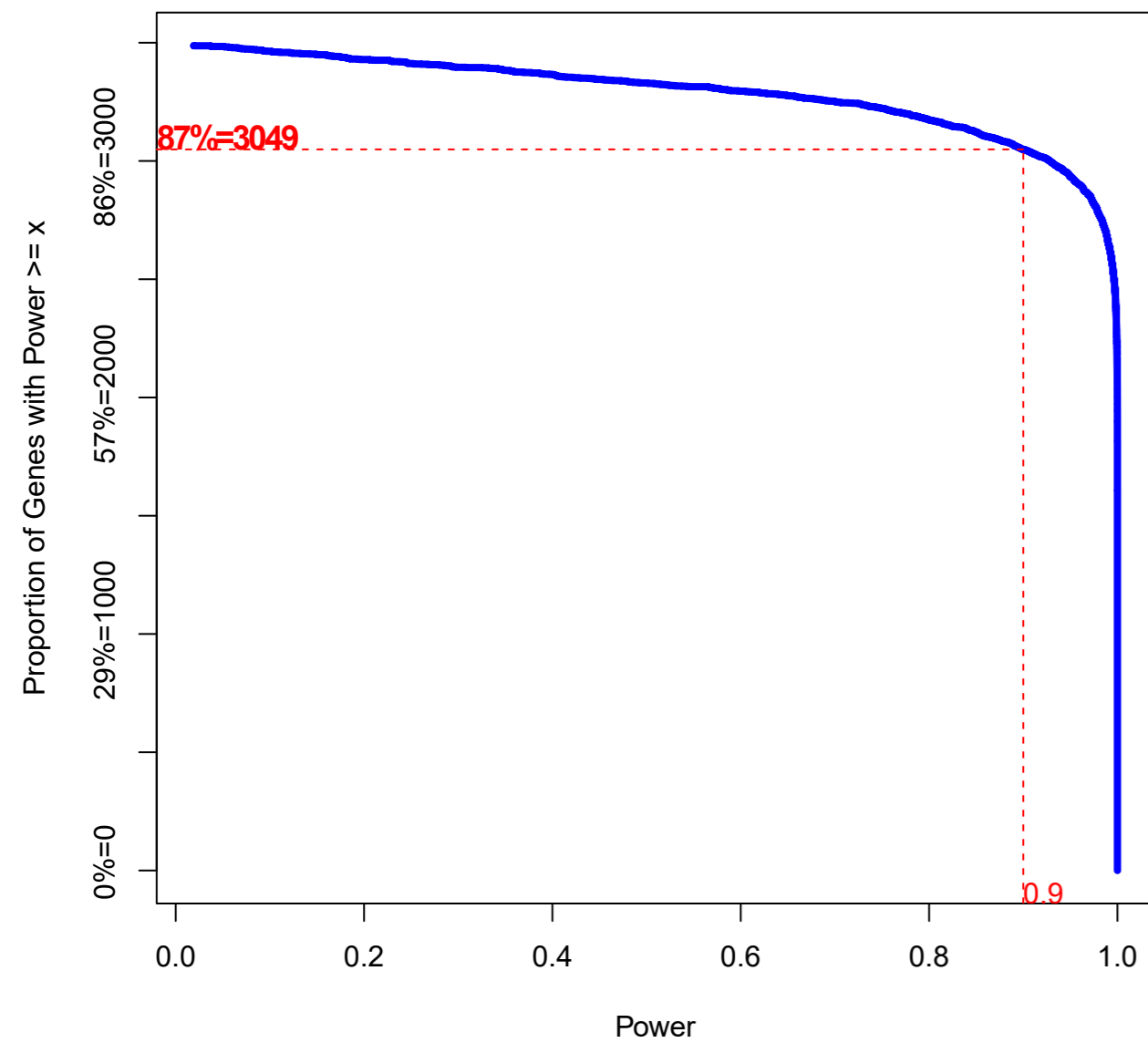

CI1/CI3

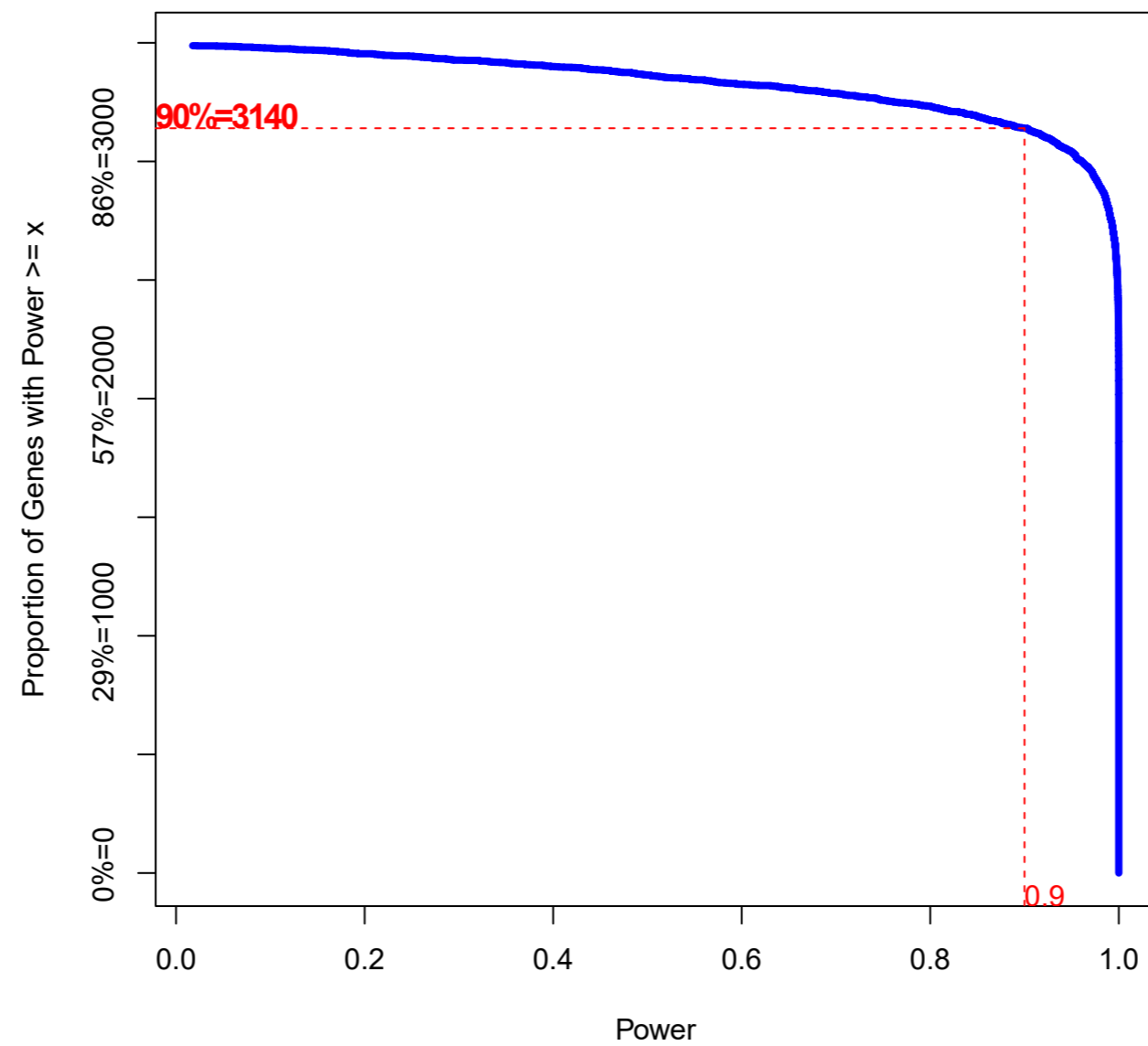

CI2/CI3

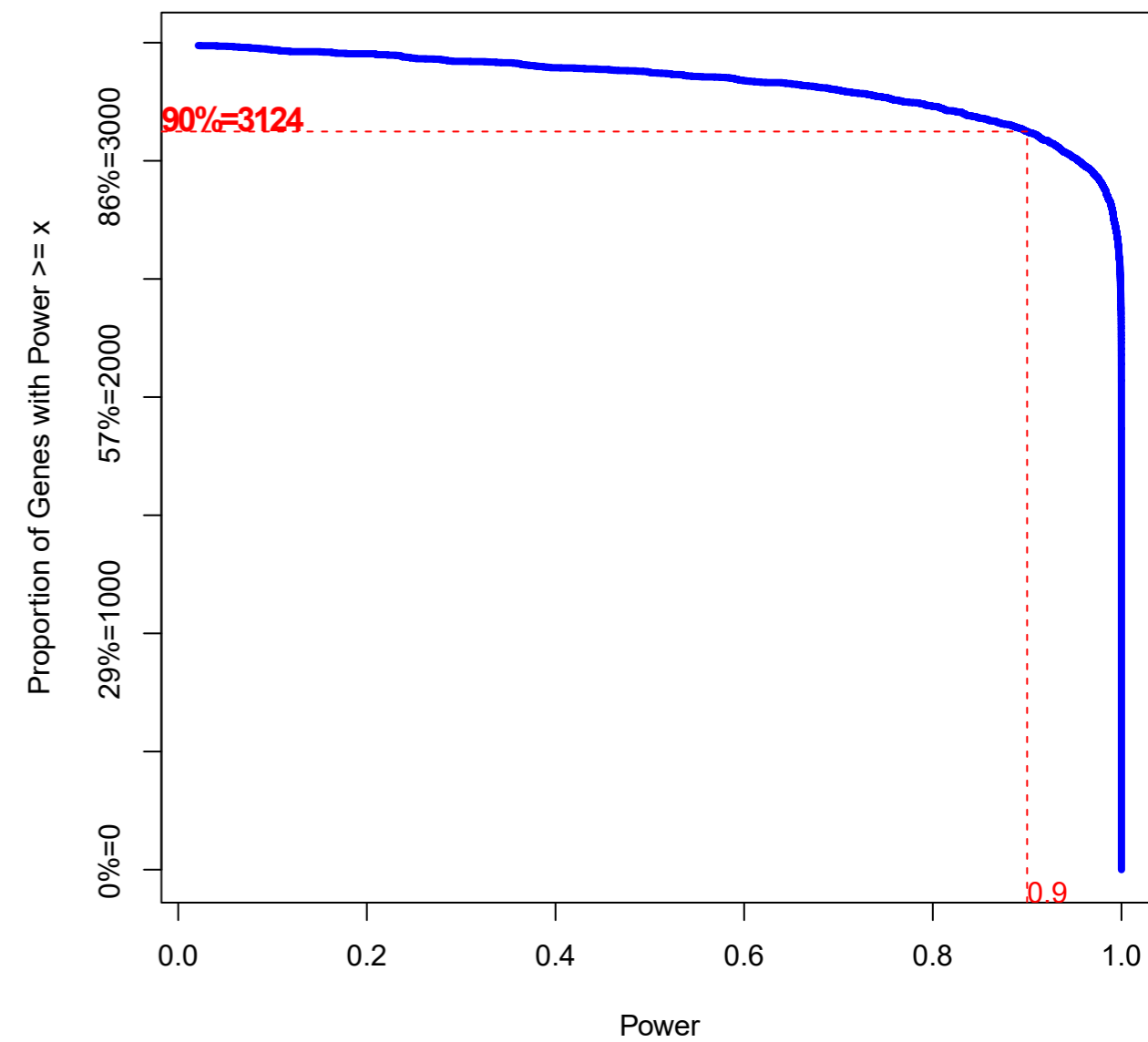

Supplement: Supplementary file 1 [file cancers-11-01057-s001.zip › cancers-558499-supplementary Materials/Supplementary Figures/FigureA1.pdf]

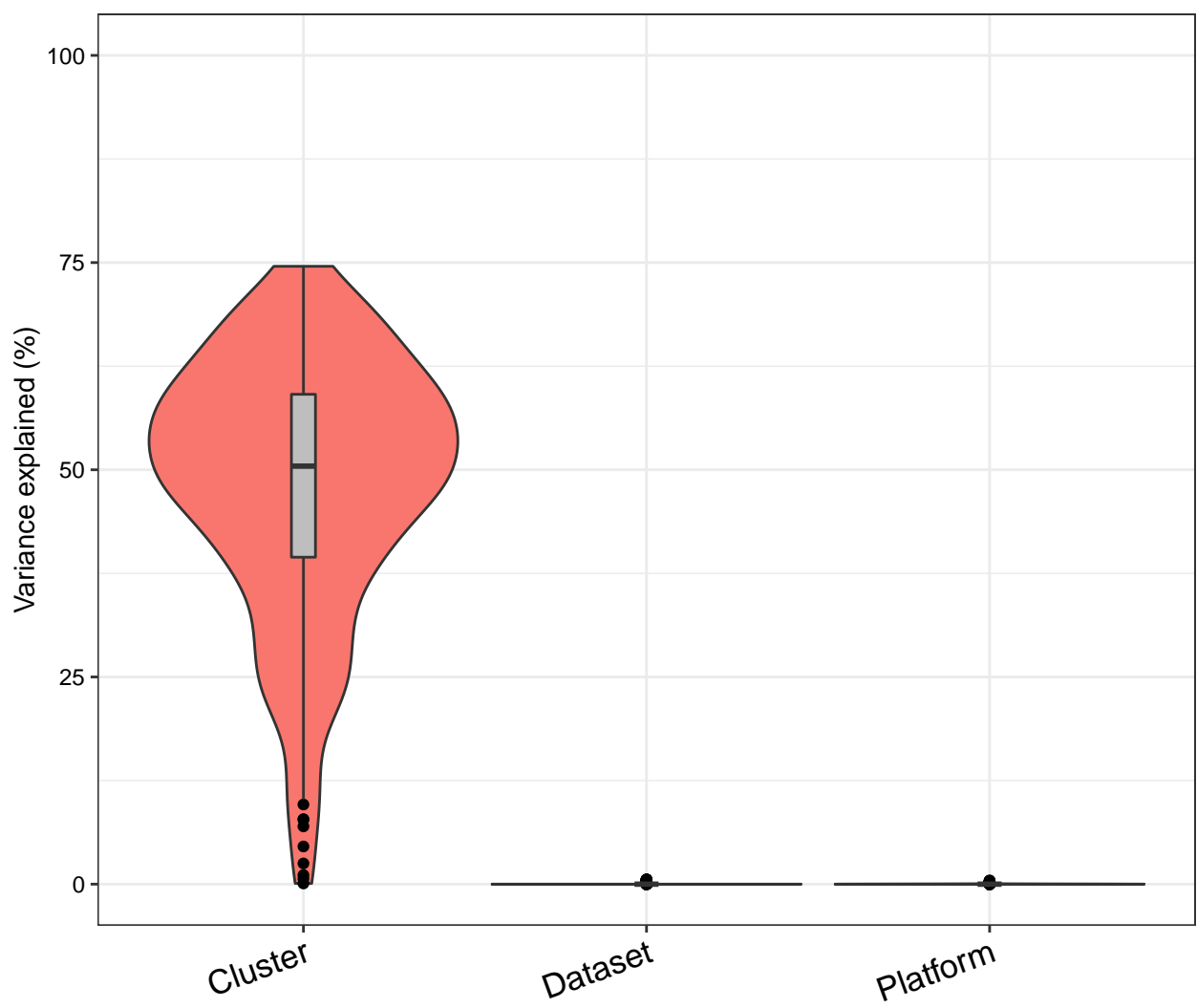

Supplement: Supplementary file 1 [file cancers-11-01057-s001.zip › cancers-558499-supplementary Materials/Supplementary Figures/FigureA2.pdf]

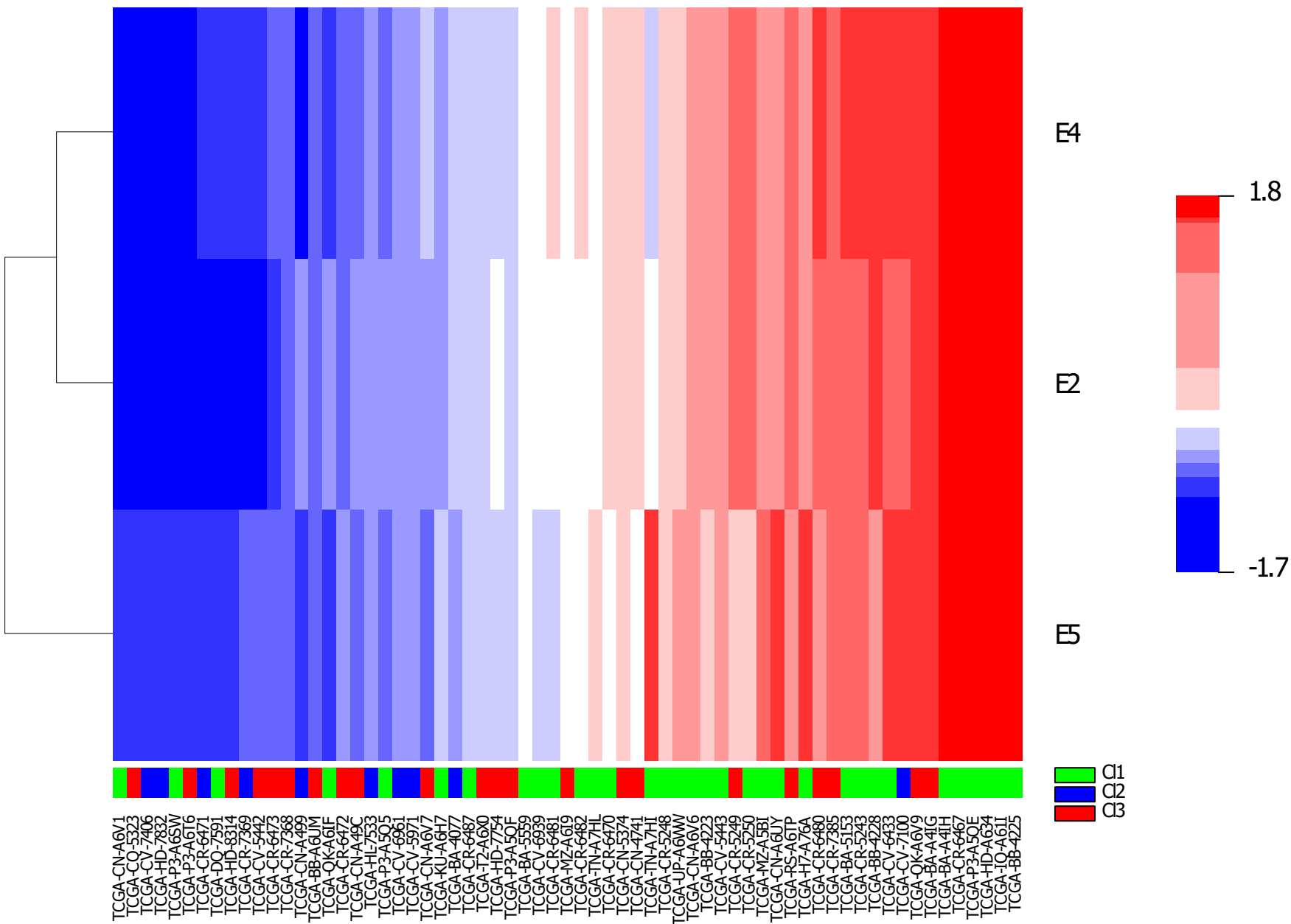

Supplement: Supplementary file 1 [file cancers-11-01057-s001.zip › cancers-558499-supplementary Materials/Supplementary Figures/FigureA3.pdf]
